# Do Highly Trained Mountain Runners Differ from Recreational Active Non-Runners on Range of Motion and Strength in the Hip and Ankle as Well as Postural Control?

**DOI:** 10.3390/jcm12072715

**Published:** 2023-04-05

**Authors:** Bartosz Zając, Maciej Olszewski, Anna Mika, Marcin Maciejczyk

**Affiliations:** 1 Laboratory of Functional Diagnostics, Central Scientific and Research Laboratory, University of Physical Education in Kraków, 31-571 Kraków, Poland; 2 Doctoral School, University of Physical Education in Kraków, 31-571 Kraków, Poland; maciejolszewski.physio@gmail.com; 3 Institute of Clinical Rehabilitation, University of Physical Education in Kraków, 31-571 Kraków, Poland; 4 Department of Physiology and Biochemistry, Faculty of Physical Education and Sport, University of Physical Education in Kraków, 31-571 Kraków, Poland; marcin.maciejczyk@awf.krakow.pl

**Keywords:** mountain running training, strength, range of motion, dynamic postural control

## Abstract

The rules governing mountain running force athletes to implement into their training programmes uphill and downhill running on unstable surfaces, which are demanding for hip and ankle as well as for the postural control system. The aim of the present cross-sectional study was to compare highly trained mountain runners (MR) and recreational active non-runners (NR) on range of motion (ROM) and strength in the hip and ankle, as well as dynamic postural control. Thirty MR and thirty-two NR were included in the study. ROM was assessed using a digital inclinometer. Strength was measured using a hand-held dynamometer. Postural control was evaluated using the lower quarter Y-balance test (YBT-LQ). The results showed that MR, in relation to NR, had statistically significant smaller hip external rotation ROM (*p* = 0.007), lower hip external rotator (*p* = 0.006) and extensor (*p* = 0.023) strength and greater normalised anterior reach in the YBT-LQ (*p* = 0.028). Mountain running training may reduce hip external rotation ROM as well as hip external rotator and extensor strength. Moreover, such training may improve postural control. MR should implement exercises targeted at developing hip ROM and strength. Furthermore, it seems that mountain running training may be a good way to improve postural control.

## 1. Introduction

Running is very common form of physical activity around the world, as demonstrated, for example, by the 15.5 million U.S. races completed in 2012 [1] and approximately 50 million people who are frequent long-distance runners in Europe [2]. Regular running provides numerous health benefits such as reduced total cardiovascular, cancer, neurological and infectious mortality [3], as well as reduced weight gain [4] and risk of cardiovascular disease [5]. However, inappropriate administration of this form of physical activity may lead to negative consequences, e.g., running-related injuries (RRI). In a meta-analysis performed by Videbæk et al. [6], it has been demonstrated that, depending on the type of runner, injury definition and length of follow-up, RRI incidence rate ranges from 2.5 to 33.0 per 1000 h of running. It should also be borne in mind that the consequences of each injury are difficulties participating in normal training and competition [7], as well as socio-economic costs [8].

One type of running, which has especially grown in popularity in recent years, is mountain running [9]. The discipline of mountain running takes place on various types of natural terrain (e.g., sand, dirt roads, forest, paths, single-track footpaths, snow), and in various kinds of environments (e.g., mountains, forests, plains, deserts). Mountain races are traditionally divided into “uphill” and “up and down” types. The average altitude gain or loss can vary from 50 m to 250 m per kilometre, and a distance up to 42.2 km [10]. Those rules force athletes to implement high-volume discipline-specific exercises into their training programmes, such as uphill (UR) and downhill (DR) running, often on various unstable surfaces, which are generally very demanding on the human body. UR and DR are different from level running (LR) with regard to movement biomechanics (i.e., foot strike pattern, ground reaction forces, joint kinematics and kinetics, as well as shock impact); therefore, they may result in specific training-induced musculoskeletal system disfunctions [11]. The most common injuries sustained by long-distance runners were found to be Patellofemoral Pain Syndrome (PFPS), Achilles Tendinopathy, Iliotibial Band Syndrome (ITBS) and Medial Tibial Stress Syndrome (MTS) [12,13,14]. Moreover, mountain running is very strenuous with regard to an athlete’s postural control system due to the presence of an unstable ground combination (after which runners must run with considerable speed) and exercise-induced fatigue, which deteriorates balance [15].

Advanced specialist sport training may cause changes in muscle strength [16,17], joint range of motion (ROM) [18,19] and dynamic postural control [20,21]. On the one hand, these changes are necessary to improve performance; on the other, they may lead to dysfunction associated with RRI. Assessment of the listed variables may be performed using reliable, valid, inexpensive, portable and easy-to-use tools in a clinical setting. Hand-held dynamometers (HHD) are convenient and versatile devices used for muscle strength testing. A HHD can be placed between the hand of the practitioner and the athletes’ tested body part, similar to how a practitioner would perform a manual muscle test. Unlike manual muscle testing, HHD provides a quantified measurement of force [22]. Digital inclinometers (DI) are one of the tools used to assess ROM in the joints. The DI is an instrument applied to measure surface inclination (in degrees) by sensors sensitive to gravity. One of the significant advantages of DI in ROM measurements is that its positioning does not depend that much on anatomic references [23]. The lower quarter Y-balance test (YBT-LQ) is used to evaluate dynamic postural control. YBT-LQ is popular and extensively utilised for injury risk identification, return-to-sport testing and pre–post-intervention measurements [24].

Based on a systematic review performed by Francis et al., [14] it seems that the foot/ankle is second most frequently injured area among runners. In turn, the hip holds just fifth place in this ranking, but dysfunctions of this area are associated with most common RRI located in the knee area [25,26]. Moreover, the hip joint seems to be the most stressed during non-level running [27,28,29]. To the best of our knowledge, there are no studies on the assessment of implications in mountain running training regarding the ROM and strength of the hip and ankle, as well as postural control in highly trained athletes. These data may be valuable for physiotherapists as well as strength and conditioning coaches involved in developing exercise programmes focused on compensating the negative consequences of mountain running training and/or directed at reducing the risk of RRI in athletes practicing this type of activity. Moreover, if mountain running training turns out to improve postural control, it would be an interesting endurance alternative to strength training on unstable surfaces aimed at balance development in athletes. We hypothesise that mountain running training, in which UR, DR, unstable ground and large training loads are present, may cause deficits in hip and ankle ROM as well as strength, and may improve dynamic postural control. Therefore, the aim of the present cross-sectional study was to compare MR and NR on ROM and strength in the hip and ankle, as well as dynamic postural control.

## 2. Materials and Methods

### 2.1. Participants

The study involved 30 highly trained MRs and 32 recreationally active NRs. Basic characteristic of studied populations are showed in Table 1. The inclusion and exclusion criteria are presented in Table 2. The participants were informed about the research protocol and provided their written informed consent to participate in the study. All procedures were carried out in accordance with the 1964 Declaration of Helsinki and its subsequent amendments. Consent to perform testing was provided by the Bioethics Committee at the Regional Medical Chamber in Krakow (no. 206/KBL/OIL/2022).

### 2.2. Study Design

In this cross-sectional study carried out among highly trained mountain runners (MR) and recreational active non-runners (NR), the following variables were bilaterally assessed: ankle dorsiflexion (DF) ROM, internal (IR) and external (ER_R_) hip rotation ROM and hip abductor (ABD), extensor (EXT) and external rotator (ER_S_) strength, as well as dynamic postural control. A participant’s stance leg was determined as the opposite of their self-preferred leg for kicking a ball [31]. Moreover, at baseline, anthropometric measurements were taken. Each measurement was carried out without blinding by one researcher (physiotherapist with 4 years’ experience), without a warm-up, between 8:00 a.m. and 12:00 a.m. Every participant was instructed not to consume stimulants (e.g., caffeine) and/or alcohol on the test day and not to perform heavy endurance or strength exercise 36–48 h prior to testing.

### 2.3. Range of Motion Measurements

ROM assessment was performed using the Baseline digital inclinometer (RMS UK Ltd., London, UK). The mean of 3 measurements was considered for analysis. Ankle DF ROM was measured during the Weight-Bearing Lunge Test, using the protocol described by Bennell et al. [32]. The participant stood facing the wall with his hands on it. Each subject was instructed to maximally move the knee forward without lifting the heel off the ground. The inclinometer was placed 15 cm below tibial tuberosity to define maximal shank inclination. Passive hip IR and ER_R_ ROM were measured following the protocol proposed by Carvalhais et al. [33]. The participant was in a prone position on the treatment table with the knee flexed to 90°. The participant’s pelvis was additionally stabilised by binding to the table using a rigid strap to minimise lumbopelvic compensatory movements during hip rotations. The examiner performed passive hip rotation until he noted tension of the muscles or passive structures of the hip joint stopped this movement. The inclinometer was placed on the lateral shank side for external hip rotation and on the medial shank side for the internal hip.

### 2.4. Strength Measurements

Isometric strength assessment was performed using the MicroFET2 hand-held dynamometer (Hoggan Health Industries Inc., West Jordan, UT, USA). The participant’s pelvis was stabilised by binding to the table using a rigid strap to minimise lumbopelvic compensatory movements during the trials. The participants performed 3 maximal isometric contractions for 5 s each with a 15 s rest between trials. The mean of 3 measurements was considered for analysis. The average force value was multiplied by the force arm length and normalised to the body mass (Nm × kg^−1^). Arm of force measurements were performed using measuring tape (TK Gruppe Klingler, Hong Kong, China). Body mass was determined using the MC 780 MA analyser (Tanita, Japan). ER_S_ hip strength was evaluated in the prone position with 90° knee flexion using the protocol described by Mendonça [34]. The dynamometer was placed proximally to the medial malleolus. Force arm length represents the linear distance from dynamometer placement to hip axis of rotation. EXT hip strength was measured in the prone position with the knee flexed to 90° according to Thorborg et al. [35]. The dynamometer was placed 5 cm proximal to the knee joint line on the posterior thigh. Force arm length represents the linear distance from dynamometer placement to the greater trochanter. ABD hip strength was measured using the protocol described by Bittencourt et al. [36] in the side-lying position. The dynamometer was placed proximally to the lateral femoral condyle. Force arm length represents the linear distance from dynamometer placement to the greater trochanter.

### 2.5. Dynamic Postural Control Assessment

Dynamic postural control was assessed using the YBT-LQ. Each participant performed 6 training and 3 testing trials in each direction (ANT, PL, PM) for both stance (SL) and kicking leg (KL) using the Y-balance test kit (Move2Perform, Evansville, IN, USA) [37]. Training and testing trials were separated by a 1 min rest. Prior to performing the trials, all participants received verbal instruction and visual demonstration. Each participant performed the single-leg stance barefoot on a starting block. They used the opposite leg as the reaching one to push the reach indicator box as far as possible in the following directions and order: anterior (ANT), posteromedial (PM) and posterolateral (PL). Attempts did not count if the participant was unable to maintain single-leg balance throughout movement, kicked the indicator box or did not return to centre position with maintained balance. The mean of 3 testing trials in each direction was used for further analysis. Scores were calculated by dividing the average reach distance (in cm) by the participant’s leg length, which was measured using measuring tape (TK Gruppe Klingler, China) in the supine position from the anterior superior iliac spine to the medial malleolus [38]. To calculate the composite score sum of average reaches in each of the 3 directions, the value was divided by 3 leg lengths and multiplied by 100%. Normalised reach distance was calculated as the average reach distance divided by the leg length and multiplied by 100% [39].

### 2.6. Statistical Analysis

Statistical analysis was carried out using Statistica 13.3 software (TIBCO Software Inc., Palo Alto, CA, USA). The differences between groups in the measured variables were evaluated with the independent sample *t*-test, the Cochran–Cox test or the Mann–Whitney U test, depending on variable distribution and equality of variance. The variable distribution in groups was examined with the Shapiro–Wilk test. In turn, the equality of variable variance between groups was assessed using Levene’s test. The probability of type I error below 0.05 was adopted as the level of significance. The test power was calculated post hoc using G*Power 3.1.9.6 software (Franz Faul, Universität Kiel, Kiel, Germany).

## 3. Results

### 3.1. Range of Motion

MRs had significantly smaller hip ER_R_ ROM in relation to NRs (Table 3).

### 3.2. Strength

MRs had significantly lower hip ER_S_ and EXT strength in relation to NRs (all differences registered for KL). Moreover, MR characterised the KL/SL ratio for ER_S_ strength closer to 1 than NR (Table 4).

### 3.3. Dynamic Postural Control

MRs obtained substantially greater normalised ANT reach than NRs (Table 5).

## 4. Discussion

The aim of the present cross-sectional study was to compare MR and NR on ROM and strength in the hip and ankle, as well as dynamic postural control. The most important finding from our study is that mountain running training may reduce hip ER_R_ ROM as well as ER_S_ and EXT strength. What is also of significance in this research is that mountain running training may, to a certain extent, improve dynamic postural control. To the best of our knowledge, this is the first study in which the effects of mountain running training on strength and range of motion in the hip and ankle were examined, as well as postural control, in highly trained athletes.

Our results suggest that mountain running training may reduce hip ER_R_ ROM, as well as ER_R_ and EXT hip strength. Interestingly, statistically significant differences were registered only for the kicking leg. Moreover, the results indicated that MRs, in relation to NRs, are more symmetrical (inter-limb ratio closer to 1) in terms of hip ABD and ER_R_ strength. In the literature, there are few studies in which the impact of running training has been assessed in the hip area. In the research by Cannon et al. [19], distance runners and non-runners (collegiate students) were compared in terms of IR and ER_R_ ROM, which was measured both at 0° and 90° hip flexion. Runners in this study showed greater IR ROM (50.82 ± 10.92° vs. 42.58 ± 6.88°) at 0° of flexion. In turn, the results derived from Hollman’s study [18] reported lower hip ER_R_ ROM among runners (33.1 ± 5.3°) compared to non-runners (40.3 ± 4.4°)—measurement performed in sitting position (90° of hip flexion). The lower values of hip ROM and strength for the MRs obtained in our study may have resulted from exposure to UR and DR, the biomechanics of which are very demanding on the hip joint. The results of a laboratory study performed by DeVita et al. [27] suggest that during UR, the hip joint seems to be the most stressed when positive work is considered. Telhan et al. [28] showed that hip power generation increases during UR. In turn, power absorption by this joint increases during DR in relation to LR. Similar results were obtained by Roberts and Belliveau [29] who found that the net work done by the hip increased with inclined running (by approximately 140% at a 21.1% grade in relation to LR). Moreover, greater hip ROM during the braking phase in DR [40] and greater hip flexion at foot strike in UR [41] were observed compared to LR. It should also be borne in mind that endurance runners apply large training volumes (up to 500 h per year [42]), meaning they are exposed to these specific loads for prolonged periods. Furthermore, it was shown that passive hip ROM decreases with increasing age [18,43]. In our study, groups were not homogenous in terms of age; therefore, this could have potentially enhanced the observed differences. We suggest that reduced hip ER_S_ and EXT strength in MRs is not a positive phenomenon. In some studies, it has been indicated that it is associated with an increase in RRI [44]. In turn, it is ambiguous whether reduction in ROM is beneficial or harmful because it may have implications for running economy [45,46]. For that reason, it should be clinically considered.

In this study, it was shown that MRs have a greater normalised ANT reach in the YBT-LQ for the kicking leg with regard to NRs. At this point, it is also worth noting that the DF ROM ankle, which is considered one of the key determinants of performance in the ANT reach [47], did not differ significantly between the groups. Therefore, we suggest that mountain running training may, to a certain extent, improve dynamic postural control. Greater normalised reach only in the ANT direction is probably due to the fact that mountain runners must often cover sections of a positive slope. Khassetarash et al. [48] showed that ankle and knee range of motion (i.e., difference between the maximal and minimal joint angle during stance phase) throughout uphill running is greater in relation to level running. Another explanation is simply that runners generally move in the sagittal plane. The probable factor causing dynamic balance improvement is the unstable surfaces present during training and competitions, which require strong engagement of the postural control system. In a meta-analysis performed by Behm et al. [49], it was shown that strength training on an unstable surface, as compared with control conditions (i.e., no training or regular training only), improved balance in healthy adolescents, young adults and older adults when tested on a stable surface (standardised mean difference of 1.18 [95%CI: 0.22–2.14]). Perhaps endurance training on an unstable surface (e.g., mountain running) is an equally good way to improve dynamic postural control.

It needs to be noted that the individual participant inter-limb ratio is considered a risk factor for sports-related injuries when limbs differ more than 10–15% [50]. These values will vary depending on the type of sport and, in particular, whether or not there is a limb dominance [51]. Kambayashi et al. [52] found that hip ABD and ER_S_ strength can independently predict noncontact anterior cruciate ligament injury in both male and female competitive athletes. It means that the strength of these muscle groups may be crucial in terms of screening and injury prevention among athletes. The results from our study suggest that MRs are less exposed to inter-limb asymmetry, probably due to symmetrical and repetitive movement patterns which occur during running. Therefore, if high values of lower-limb strength asymmetry are observed in individual runners, it is worth looking for the cause of their formation and applying corrective interventions.

Our results also have several practical applications. Existing hip ROM and strength deficits in the MR group may require additional interventions. We suggest complementary training or physical therapy, which should include elements of increasing hip ER_R_ ROM as well as strength training of the hip musculature. Apart from specific targeted interventions, exercise programmes should also integrate foot and hip function to activate the entire kinetic chain in functional positions that simulate running [53]. However, to this day, there are no studies evaluating the effects of these particular interventions in the MR population. There is still a need to understand the mechanisms of musculoskeletal adaptations in the hip area to ascertain whether the observed changes are positive or harmful. On the other hand, we found that MR training can possibly improve dynamic postural control in the sagittal plane. This could be beneficial for athletes implementing running training on unstable surfaces and changing slope levels as a method of increasing dynamic balance, especially among individuals with noticeable deficits in the YBT-LQ ANT reach.

The limitations of the study include: (i) lack of a “classic” runner group for comparison; therefore, we do not know if the obtained results are characteristic only for mountain runners or for all runners; (ii) non-homogeneity of groups in terms age, body mass and physical performance; (iii) performing research on a homogeneous group (in terms of sex), meaning the results cannot be generalised to female runners. In order to validate the injury risk predictive value of the measurements used in this study, there is a need to verify in future research the internal validity of the evaluated outcomes.

## 5. Conclusions

Mountain running training may reduce hip ER_R_ ROM, as well as ER_S_ and EXT hip strength. Moreover, mountain running training may, to a certain extent, improve dynamic postural control. Therefore, MRs should implement exercise targeted at developing ER_R_ ROM and hip ER_S_, as well as EXT strength. Furthermore, it seems that mountain running training may be a good way to improve dynamic postural control in athletes. In future studies, a comparison of mountain runners and “classic” distance runners should be considered in terms of ROM and strength of the hip and ankle. In addition, in the future, a comparison of endurance training on an unstable surface and strength training on an unstable surface in terms of effectiveness in dynamic postural control improvement among athletes should be conducted.

## Figures and Tables

**Table 1:** Basic characteristics (mean (SD)) of the studied populations.

	Mountain Runners	Non-Runners	*p*	Test Power
Age (years)	28.5 (6.6)	21.8 (2.5)	0.000 *^,M-W^	0.99
Body height (cm)	179.1 (7.4)	177.2 (7.0)	0.315 ^T^	0.18
Body mass (kg)	71.5 (7.4)	79.6 (11.0)	0.001 *^,CC^	0.92
Body mass index (kg × m^−2^)	22.7 (1.6)	24.8 (2.9)	0.001 *^,CC^	0.94
Training experience (years)	8.4 (3.3)	NA	NA	NA

*p*—probability of type I error, *—statistically significant difference, ^M-W^—Mann–Whitney U test, ^T^—*t*-test, ^CC^—Cochran–Cox test, SD—standard deviation, NA—not applicable.

**Table 2:** Inclusion and exclusion criteria for the study.

	Mountain Runners	Non-Runners
Inclusion criteria	Age: 18–40 years Sex: male	
	Tier: Highly trained/national-level ^#^ Minimum 3 years of training experience	Tier: Recreationally active ^#^
Exclusion criteria	Lower-limb massive injuries in the past (anterior cruciate ligament rupture, hip fractures, instability and recurrent ankle sprains) Minor lower limb injury in past 3 months before examination that may disable performance on the Lower Quarter Y-balance test Diagnosed difficulties in maintaining balance Diagnosed ankle, knee or hip instability	

^#^ Tier classification was performed according to McKay et al. [30].

**Table 3:** Results (mean (SD)) of ROM measurement.

	Mountain Runners	Non-Runners	*p*	Test Power
Ankle dorsiflexion ROM (°)				
Kicking leg	42.8 (4.8)	42.3 (5.4)	0.692 ^T^	0.07
Stance leg	42.7 (5.3)	42.8 (4.4)	0.920 ^T^	0.05
Kicking/stance leg ratio	1.00 (0.11)	0.99 (0.83)	0.658 ^T^	0.05
Hip internal rotation ROM (°)				
Kicking leg	25.0 (10.8)	25.6 (9.3)	0.794 ^T^	0.06
Stance leg	36.6 (10.9)	37.5 (11.9)	0.761 ^T^	0.06
Kicking/stance leg ratio	0.67 (0.20)	0.68 (0.18)	0.779 ^T^	0.05
Hip external rotation ROM (°)				
Kicking leg	57.7 (10.7)	64.8 (9.5)	0.007 *^,CC^	0.79
Stance leg	41.6 (10.5)	47.1 (11.9)	0.054 ^T^	0.49
Kicking/stance leg ratio	1.43 (0.25)	1.42 (0.24)	0.965 ^T^	0.05

ROM—range of motion*, p*—probability of Type I error, *—statistically significant difference, ^T^—*t*-test, ^CC^—Cochran–Cox test.

**Table 4:** Results (mean (SD)) of strength measurement.

	Mountain Runners	Non-Runners	*p*	Test Power
Hip external rotation strength (Nm × kg^−1^)				
Kicking leg	0.85 (0.10)	0.94 (0.14)	0.006 *^,CC^	0.83
Stance leg	0.84 (0.13)	0.87 (0.15)	0.345 ^T^	0.13
Kicking/stance leg ratio	1.02 (0.05)	1.08 (0.10)	0.003 *^,CC^	0.85
Hip extensor strength (Nm × kg^−1^)				
Kicking leg	1.05 (0.20)	1.19 (0.26)	0.023 *^,CC^	0.66
Stance leg	1.02 (0.23)	1.08 (0.28)	0.350 ^T^	0.15
Kicking/stance leg ratio	1.05 (0.12)	1.12 (0.22)	0.088 ^T^	0.34
Hip abductor strength (Nm × kg^−1^)				
Kicking leg	1.81 (0.10)	1.89 (0.29)	0.320 ^T^	0.31
Stance leg	1.82 (0.33)	1.80 (0.29)	0.825 ^T^	0.06
Kicking/stance ratio	1.00 (0.05)	1.06 (0.14)	0.028 *^,CC^	0.61

*p*—probability of Type I error, *— statistically significant difference, ^T^—*t*-test, ^CC^—Cochran–Cox test.

**Table 5:** Lower quarter Y-balance test performance.

	Mountain Runners	Non-Runners	*p*	Test Power
Anterior reach (%)				
Kicking leg, mean (SD)	68.4 (6.3)	64.9 (6.4)	0.028 *^,CC^	0.58
Stance leg, mean (SD)	67.4 (5.8)	66.2 (6.2)	0.414 ^T^	0.12
Kicking/stance ratio, median (Q_1_–Q_3_)	1.01 (0.97–1.05)	0.98 (0.95–1.00)	0.005 *^,M-W^	0.71
Posteromedial reach (%)				
Kicking leg, mean (SD)	116.0 (8.1)	114.0 (7.7)	0.403 ^T^	0.17
Stance leg, mean (SD)	116.0 (6.7)	115.0 (7.4)	0.440 ^T^	0.09
Kicking/stance ratio, median (Q_1_–Q_3_)	1.00 (0.97–1.01)	0.99 (0.97–1.02)	0.979 ^M-W^	0.05
Posterolateral reach (%)				
Kicking leg, mean (SD)	113.0 (7.7)	111.0 (8.5)	0.328 ^T^	0.16
Stance leg, mean (SD)	113.0 (8.0)	111.0 (8.5)	0.334 ^T^	0.16
Kicking/stance ratio, mean (SD)	1.00 (0.05)	1.00 (0.04)	0.741 ^T^	0.05
Composite score (%)				
Kicking leg, mean (SD)	99.1 (6.5)	96.7 (6.8)	0.153 ^T^	0.29
Stance leg, mean (SD)	98.7 (5.8)	97.2 (6.6)	0.327 ^T^	0.16
Kicking/stance ratio, median (Q_1_–Q_3_)	1.00 (0.98–1.02)	1.00 (0.97–1.01)	0.270 ^M-W^	0.05

*p*—probability of Type I error, *—statistically significant difference, ^M-W^—Mann–Whitney U test, ^T^—*t*-test, ^CC^—Cochran–Cox test, SD—standard deviation, Q_1_–Q_3_—1st and 3rd quartiles.

## Data Availability

All data are available from the corresponding author on request.
